# Estradiol rescues male hydroxyl radical-mediated Charcot-Marie-Tooth 2Z by *Morc2a* stabilization through autophagy inhibition in a murine model

**DOI:** 10.1007/s00401-025-02922-2

**Published:** 2025-08-04

**Authors:** Ji Won Kim, Soo Hyun Nam, Geon Seong Lee, Hye Yoon Chung, Eun Young Kim, Jeong Pil Han, Jae-Hyung Jang, Byung-Ok Choi, Su Cheong Yeom

**Affiliations:** 1 Graduate School of International Agricultural Technology and Institute of Green BioScience and Technology, Seoul National University, 1447 Pyeongchang-Ro, Daewha, Pyeongchang, 25354 Gangwon Korea; 2 Cell & Gene Therapy Institute, Samsung Medical Center, Seoul, 06351 Korea; 3 Department of Health Science and Technology, Samsung Advanced Institute for Health Sciences & Technology, 81, Seoul, 06351 Korea; 4 Department of Neurology, Samsung Medical Center, Sungkyunkwan University School of Medicine, 81 Irwonr-Ro, Gangnam, Seoul, 06351 Korea; 5 Department of Chemical and Biomolecular Engineering, Yonsei University, 50 Yonsei-Ro, Seodaemun-Gu, Seoul, 03722 Korea; 6 GluGene Therapeutics, Seoul, 03722 Korea; 7 Department of Agricultural Biotechnology, WCU Biomodulation Major, Seoul National University, 1 Gwanak-Ro, Gwanank, Seoul, 08826 Korea

**Keywords:** Charcot-Marie-Tooth type2Z, Estradiol, Hydroxyl radical, MORC2 haploinsufficiency, Neuromuscular regeneration

## Abstract

Charcot–Marie–Tooth disease type 2Z (CMT2Z) is an inherited axonal neuropathy caused by haploinsufficiency of microrchidia CW-type zinc finger protein 2 (*MORC2*), which leads to elevated hydroxyl radical levels, reduced ATPase activity, and apoptosis-mediated neuromuscular degeneration. CMT2Z presents with severe clinical manifestations, yet no widely applicable and affordable treatment has been developed. While gene therapy presents a theoretical solution, its feasibility remains constrained by prohibitive costs and delivery challenges. We observed sex-specific differences in muscle function in a CMT2Z mouse model carrying the microrchidia CW-type zinc finger protein 2A (*Morc2a*) p.S87L variant, with males exhibiting more severe weakness, suggesting a protective role of estradiol in females. Thus, we hypothesized that identifying and utilizing this factor could contribute to CMT2Z drug development. We found that estradiol stabilizes the *Morc2a* variant protein by inhibiting autophagy, independently of specific estrogen receptors, thereby mitigating hydroxyl radical–induced mitochondrial aggregation and apoptosis while restoring ATPase function. Subcutaneous implantation of estradiol pellets in the CMT2Z mouse model significantly improved *Morc2a* protein stability in the *quadriceps femoris* and sciatic nerve, reversed mitochondrial aggregation, and ameliorated both muscular and peripheral nerve degeneration. Notably, symptomatic *Morc2a* p.S87L mice exhibited robust peripheral nerve regeneration, demonstrating estradiol’s ability to restore function rather than merely delay disease progression. Moreover, the therapeutic effects were reproduced in human *MORC2* p.R252W variants, further confirming its translational potential. As an FDA-approved compound with well-characterized pharmacokinetics, estradiol represents a rapidly deployable strategy for treating CMT2Z. This study highlights the pivotal role of oxidative stress in the pathophysiology of CMT2Z and identifies MORC2 stabilization as a promising intervention. Moreover, the findings advocate for repurposing existing therapeutics to address rare genetic disorders, broadening treatment paradigms for neuromuscular diseases beyond CMT2Z.

**Supplementary Information:**

The online version contains supplementary material available at 10.1007/s00401-025-02922-2.

## Introduction

Charcot-Marie-Tooth (CMT) disease is a hereditary neuropathy affecting the peripheral nervous system [43], leading to muscle weakness, sensory loss, and deformities such as *pes cavus* and hammer toes [25]. The disease is caused by genetic mutations and is classified into multiple subtypes based on the site of nerve damage and the mode of inheritance. The most common form, CMT type 1A, results from a mutation in the Peripheral Myelin Protein 22 (*PMP22*) gene, leading to demyelination, whereas CMT type 2 encompasses axonal neuropathies caused by diverse genetic mutations [6]. Despite advances in understanding CMT pathogenesis, effective treatments remain unavailable. Current management strategies, including physical therapy, occupational therapy, assistive devices, pain management, and surgical interventions, are palliative rather than curative [24]. Several barriers hinder the development of targeted therapies, including the limited regenerative capacity of neurons, the complexity of CMT pathophysiology, diagnostic challenges, the absence of reliable animal models, and difficulties in achieving efficient drug delivery to the nervous system.

Microrchidia CW-type zinc finger protein 2 (*MORC2)* functions as a chromatin remodeling factor involved in the DNA damage response, transcriptional repression, and epigenetic regulation, and its precise activity is essential for preserving neuronal integrity [44, 49]. *MORC2* cause diverse disorders depending on the location of the single nucleotide polymorphism, including axonal neuropathy (CMT2Z), spinal muscular atrophy (SMA), developmental delay, impaired growth, dysmorphic facies, axonal neuropathy (DIGFAN), and cancer [37]. In previous studies, we developed a mouse model carrying the microrchidia CW-type zinc finger protein 2A (*Morc2a*) p.S87L mutation (C56BL/6.*Morc2a*^em(S87L)^, *Morc2a* p.S87L) and demonstrated axonal degeneration mediated by cellular apoptosis [19]. Both *Morc2a* p.S87L and p.R252W variants reduced *Morc2a* protein stability, resulting in haploinsufficiency. This haploinsufficiency increased hydroxyl radical (HO•) levels and decreased nuclear ATPase activity, ultimately inducing cellular apoptosis [9].

A fundamental approach to treating genetic disorders is gene correction. However, gene-editing materials face significant delivery challenges in neurons, resulting in low efficiency and leaving uncorrected cells susceptible to apoptosis. An alternative therapeutic strategy for conditions involving loss of function or haploinsufficiency is recombinant adeno-associated virus (rAAV)-mediated gene therapy. This approach has led to Food and Drug Administration (FDA)-approved gene therapies for neuromuscular disorders such as spinal muscular atrophy and Duchenne muscular dystrophy, with several other candidates undergoing clinical trials [35]. Among the viral vectors, AAV PHP.eB offers an efficient gene delivery platform, enabling widespread distribution within the nervous system in mice, which is particularly advantageous for treating CMT-related neuropathies [34]. In a previous study, we demonstrated that AAV PHP.eB-mediated delivery of the Microrchidia CW-Type Zinc Finger 2A (*Morc2a*) GHKL ATPase domain reduced apoptosis in neuronal and muscular tissues while restoring normal functional levels [9]. However, despite the promise of gene therapy, its widespread implementation is constrained by prohibitive costs, with FDA-approved gene therapies ranging from $800,000 to $3 million per treatment [10]. Furthermore, like many neuromuscular genetic disorders, CMT2Z is a rare disease, and ensuring the economic feasibility of gene therapy development is particularly challenging [11].

Among the CMT2Z-associated pathogenic mechanisms, *Morc2a* haploinsufficiency leads to both reduced ATPase activity and excessive hydroxyl radical formation, with cellular apoptosis in CMT2Z primarily driven by oxidative stress. Thus, an initial therapeutic approach could involve targeting hydroxyl radicals. The hydroxyl radical scavenger nicaraven consistently exhibited therapeutic effects in CMT2Z animal models [9]. Although several hydroxyl radical scavengers and inhibitors have been explored [12], none have received FDA approval for clinical use. Additionally, reduced ATPase activity in *Morc2a* p.S87L may impair DNA repair, further contributing to neuropathic progression [15]. Unlike strategies solely aimed at hydroxyl radical inhibition, approaches that restore Microrchidia Family CW-Type Zinc Finger 2 (MORC2) protein expression could address both ATPase deficiency and oxidative stress, thereby providing a more comprehensive therapeutic solution. Potential strategies of MORC2 restoration include (1) transcriptional activation of *MORC2* and (2) stabilization of MORC2 protein.

Therapeutic strategies can also be guided by phenotypic analysis of patients and animal models. Our *Morc2a* p.S87L mice muscle function assessment revealed sex-specific differences, with males exhibiting more severe muscular weakness. This finding suggested the presence of a protective factor in females that mitigates muscle-related symptoms, leading us to hypothesize that identifying and utilizing this factor could contribute to CMT2Z drug development. Through this investigation, we identified estradiol as a key factor associated with symptom alleviation in female mice and elucidated its mechanism in counteracting *Morc2a* haploinsufficiency. Further studies demonstrated that estradiol administration in disease models significantly improved neuromuscular function. This research suggests that an estradiol-based therapeutic approach could be rapidly and cost-effectively implemented in CMT2Z patients, given that its dosage, pharmacokinetics, distribution, and safety profile have already been extensively characterized.

## Materials and methods

### Animal

Following anesthesia, wild-type (WT) or *Morc2a* p.S87L mice were euthanized, and tissues were harvested for analysis. Organs were subsequently processed according to experimental requirements—either snap-frozen at − 80 °C, fixed in formalin, or embedded in OCT compound for cryosectioning. All animal procedures were approved by the Institutional Animal Care and Use Committee (IACUC) of Seoul National University (Approval No. SNU-241108–6) and conducted in strict accordance with institutional ethical guidelines.

### Western blot analysis

For protein extraction, tissues or chemically treated cells were lysed on ice for 30 min using either NP-40 buffer (Cat. No. 85125; Thermo Fisher Scientific, Waltham, MA, USA) or T-PER buffer (Cat. No. 78510; Thermo Fisher Scientific), each supplemented with protease inhibitors. Lysates were clarified by centrifugation at 12,000 × g for 15 min at 4 °C, and protein concentrations were determined using the BCA Protein Assay Kit (Thermo Fisher Scientific). Equal amounts of protein were resolved by SDS-PAGE and transferred onto polyvinylidene fluoride (PVDF) membranes (EMD Millipore, Billerica, MA, USA) via electrotransfer. Membranes were blocked with 5% (w/v) skim milk in Tris-buffered saline (TBS) for 1 h at 25 °C and incubated with primary antibodies at 4 °C for 24–48 h. After washing with TBS containing 0.1% Tween-20 (TBS-T), membranes were incubated with horseradish peroxidase (HRP)-conjugated secondary antibodies for 1 h at 25 °C. Protein bands were detected using an enhanced chemiluminescence (ECL) kit (Abclon, Seoul, Korea) and imaged with a Fusion Solo S system (Vilber, Collégien, France). Band intensities were quantified using ImageJ (NIH, Bethesda, MD, USA). Chemiluminescent Western blot images were exported and converted to 8-bit grayscale. Rectangular regions of interest (ROIs) encompassing each lane were defined, and the densitometric peak area corresponding to the target band was measured. Each target signal was normalized to the β-actin or α-tubulin band intensity from the same lane. Normalized values were expressed relative to the control group. Information regarding primary and secondary antibodies, including host species, dilution ratios, and clone numbers, is provided in Supplementary Table 1.

### Grip strength measurement

Hind limb grip strength was assessed using a grip strength meter (BIO-GS3; Bioseb, Vitrolles, France). Mice were allowed to grasp a T-bar with their hind paws and were gently pulled backward along a horizontal plane. The peak force exerted before the grip was released was recorded as the maximal tension. Each mouse performed three trials at 20 min intervals, and the average of the three values was used for analysis.

### Clinical examination

Clinical data were obtained from the most recent assessment of the affected individuals as previously described [31]. Muscle strength of the flexor and extensor groups was manually assessed using the standard Medical Research Council (MRC) scale. Functional disability was assessed using two standardized instruments, the Functional Disability Scale (FDS) and the Charcot-Marie-Tooth Neuropathy Score version 2 (CMTNSv2). Sensory deficits were assessed according to the degree of impairment of pain, temperature, vibration, and proprioception. The age at onset of neurological symptoms was determined by the patient’s self-reported age at symptom appearance.

### Patients and ethical statements

This study involved members of a family harboring *MORC2* variants, including two clinically affected individuals. Written informed consent was obtained from all participants prior to enrollment. The study protocol was approved by the Institutional Review Board of Samsung Medical Center, Sungkyunkwan University (IRB No. 2014–08-057–002).

### Measurement of reactive oxygen species

To assess mitochondrial superoxide levels, MitoSOX™ Red Superoxide Indicator (MSR) reagent stock and working solutions were prepared according to the manufacturer’s instructions (Cat. No. M36008; Thermo Fisher Scientific). Cells were seeded in 96-well plates and treated with 100 µL of 5 µM MSR working solution per well. The plates were incubated at 37 °C in a 5% CO₂ atmosphere for 30 min. After incubation, the cells were gently washed three times with pre-warmed buffer. Fluorescence intensity was measured within 2 h using a Cytation 5 imaging system (BioTek). To evaluate mitochondrial hydroxyl radical levels, the culture medium was removed and replaced with 100 µL of OH580 staining solution. Following incubation at 37 °C for 1 h, cells were washed three times with either HHBS or DPBS. Subsequently, 100 µL of assay buffer was added to each well, and fluorescence intensity was measured using the Cytation 5 imaging system (BioTek).

### In vitro induction of hydroxyl radicals

Cell-based experiments were conducted with minor modifications to a previously published protocol [51]. To induce elevated levels of hydroxyl radicals, MEFs or iPSCs were treated with a combination of 25 µM FeSO₄ and 25 µM H₂O₂. After 24 h of treatment, mitochondrial superoxide and hydroxyl radical levels were assessed.

### Protein stability assay

Cells were seeded in 96-well plates at a density of 5 × 10^3^ cells per well and transfected with 0.1 µg of plasmid DNA using Lipofectamine™ 3000 (Thermo Fisher Scientific). NIH 3T3 cells were transfected with HiBiT-tagged wild-type (WT) *Morc2a* or the *Morc2a* p.S87L variant, whereas MCF7 cells were transfected with HiBiT-tagged WT *MORC2* or the *MORC2* p.R252W variant. At 24 h post-transfection, cells were treated with either 100 µg/mL cycloheximide (Cat. No. C1988; Sigma-Aldrich, St. Louis, MO, USA) to inhibit protein synthesis or 50 ng/mL bafilomycin (Cat. No. B1793; Sigma-Aldrich) to block protein degradation. In parallel, estradiol was administered every 24 h following transfection. Protein expression levels were quantified at 0, 6, 12, and 24 h post-treatment using the Nano-Glo® HiBiT Lytic Assay (Promega, Madison, WI, USA).

### ATPase activity measurement

Cells were washed and lysed using T-PER reagent (Thermo Fisher Scientific) to extract total proteins. Protein concentrations were determined using the BCA Protein Assay Kit (Thermo Fisher Scientific). ATPase activity was assessed in 96-well plates using the QuantiChrom™ ATPase/GTPase Assay Kit (DATG-200; BioAssay Systems, Hayward, CA, USA). Enzymatic activity was quantified by measuring the amount of inorganic phosphate released during the reaction, as determined by absorbance at 620 nm.

### Apoptosis analysis

Apoptosis was assessed in both cultured cells and tissue sections. For the in vitro assay, cells were stained with Annexin V-FITC and propidium iodide using the TACS Annexin V-FITC Apoptosis Detection Kit (Cat. No. 4830–250-K; R&D Systems, Minneapolis, MN, USA) and incubated at 25 °C for 15 min. FITC fluorescence was used to distinguish early and late apoptotic cells. In parallel, cryopreserved tissue sections were incubated with an FITC-conjugated Annexin V antibody (Trevigen, Gaithersburg, MD, USA) for 1 h at room temperature. Fluorescent signals indicating apoptotic regions were visualized using the Cytation 5 imaging system (BioTek).

### siRNA mediated gene inhibition

Small interfering RNAs (siRNAs) targeting *Morc2a*, *Esr1*, *Esr2*, and *Gper1* were purchased from Bioneer (Daejeon, Korea) and transfected into cells using Lipofectamine™ RNAiMAX (Thermo Fisher Scientific) according to the manufacturer’s protocol. Cells were incubated for 72 h following transfection before being used in downstream experiments. The sequences of the siRNAs employed in this study are provided in Supplementary Table 1.

### Immunohistochemistry

Paraffin-embedded tissue sections were deparaffinized in xylene and rehydrated through a graded ethanol series. Antigen retrieval was performed by heating the sections in citrate buffer (pH 6.0) at 95 °C for 20 min. After cooling to room temperature, the sections were blocked with 5% (w/v) goat serum in PBS for 90 min at 25 °C, followed by incubation with primary antibodies against cytochrome C and cleaved Caspase-3 at 4 °C for 48 h. After three washes with PBS, the sections were incubated with an HRP-conjugated secondary antibody for 1 h at 25 °C. Immunoreactivity was visualized using a DAB substrate kit (SK-4100; Vector Laboratories, Burlingame, CA, USA) and counterstained with hematoxylin. Stained sections were imaged using a light microscope.

### Immunofluorescence

Tissues were fixed in 4% paraformaldehyde (PFA) for 24 h, followed by cryoprotection in 15% and 30% sucrose solutions for 6 and 24 h, respectively. The cryoprotected tissues were embedded in OCT compound (Sakura Finetek, Tokyo, Japan) and snap-frozen at − 80 °C. Tissue blocks were sectioned at a thickness of 15 μm using a cryostat (Leica, Wetzlar, Germany). To detect reactive oxygen species (ROS), sections were incubated overnight at 4 °C with an anti-8-hydroxy-2′-deoxyguanosine (8-OHdG) antibody. Fluorescence signals were visualized using a Cytation 5 imaging system (BioTek, Winooski, VT, USA).

### Semithin and electron microscopic examination

Sciatic nerves collected from control and estradiol-treated mice were processed for pathological evaluation using both light and transmission electron microscopy. Each nerve was individually fixed in 2% glutaraldehyde prepared in 25 mM cacodylate buffer. For semi-thin sectioning, tissues were post-fixed in 1% osmium tetroxide (OsO₄) for 1 h, dehydrated through a graded ethanol series, transitioned through propylene oxide, and embedded in epoxy resin (Epon 812; Oken, Nagano, Japan). Semi-thin sections were stained with toluidine blue for light microscopy. Ultrathin sections were stained with uranyl acetate and lead citrate for ultrastructural examination. Three representative samples per group were analyzed using a bright-field microscope. For transmission electron microscopy, ultrathin sections (~ 60 nm) were mounted on 200-mesh nickel grids and post-stained with 1% uranyl acetate for 10 min, followed by Reynolds’ lead citrate. Electron micrographs were acquired using an HT7700 transmission electron microscope (Hitachi, Tokyo, Japan) operated at 80 kV. The distribution and diameter of myelinated fibers were analyzed using Zen2 imaging software (Carl Zeiss, Oberkochen, Germany). The total number of myelinated fibers per animal was quantified from montage images comprising more than 20 randomly selected fields, acquired at 600 × magnification from semi-thin sections.

### MitoTracker and mitophagy analysis

For in vitro analysis, mitochondria in MEFs were labeled with 200 nM MitoTracker Red (Cell Signaling Technology, Danvers, MA, USA) at 37 °C for 30 min. Cells were then washed twice with Dulbecco’s phosphate-buffered saline (DPBS). Mitochondrial dysfunction and mitophagy were assessed using a Mitophagy Detection Kit (Dojindo, Kumamoto, Japan). MEFs were incubated with 200 nM mitophagy dye for 1 h, followed by 1 μM Lyso dye for 30 min. After washing with DPBS, fluorescence signals were acquired using a Cytation 5 imaging system (BioTek). Cells were subsequently fixed in 4% paraformaldehyde (PFA) in PBS (Thermo Fisher Scientific) for 15 min and washed three times with DPBS. The labeled MEFs were visualized using a confocal laser scanning microscope (Leica TCS SP8; Leica). For tissue-based analysis, cryosections were incubated with 200 nM MitoTracker Red at 37 °C for 30 min and washed twice with DPBS. Nuclei were counterstained with DAPI (Thermo Fisher Scientific), and fluorescence images were acquired using the Cytation 5 imaging system (BioTek).

### Differentiation of human iPSCs from MORC2 p.R252W patient cells to motor neurons

Human dermal fibroblasts obtained from a patient with Charcot-Marie-Tooth disease type 2Z (CMT2Z), harboring the *MORC2* p.R252W mutation, were reprogrammed into induced pluripotent stem cells (iPSCs) using the CytoTune-iPS 2.0 Sendai Reprogramming Kit (Thermo Fisher Scientific). Reprogramming was performed according to the manufacturer’s instructions to induce the expression of four transcription factors: OCT3/4, KLF4, SOX2, and c-MYC. After 15 days, colonies displaying embryonic stem cell-like morphology were manually selected and expanded under feeder-free conditions using mTeSR medium. iPSCs were passaged onto Matrigel-coated 6-well plates and cultured at 37 °C in a humidified atmosphere containing 5% CO₂ until they reached approximately 80% confluency. At this stage, neural induction was initiated using a monolayer-based differentiation protocol adapted from Shi et al. [38], resulting in the generation of regionally unspecified neural progenitor cells. These cells were subsequently replated onto culture surfaces coated with 0.01% poly-L-ornithine (Sigma-Aldrich) and 5 μg/mL laminin (Sigma-Aldrich), and maintained under conditions that promote motor neuron differentiation, following the method described by Amoroso et al. [1]. This protocol consistently yielded cultures in which 30–40% of cells expressed the motor neuron marker Islet-1. To enrich for motor neurons, non-neuronal cells were depleted via magnetic-activated cell sorting (MACS) on day 25 post-induction, using antibodies against CD184 and CD44 (BD Biosciences, Franklin Lakes, NJ, USA) [47].

### Estradiol pellet transplantation

Male and female B6 and *Morc2a* p.S87L heterozygous mice aged 20 months were used in the experiments. Animals were randomly assigned to experimental groups. To administer estrogen, 90 day-release 17β-estradiol pellets (Innovative Research of America, Sarasota, FL, USA) were subcutaneously implanted into the left dorsal region of each mouse. Mice were anesthetized with isoflurane, and the surgical site was shaved. A small incision (~ 5 mm) was made in the left dorsal region, and the pellet was inserted subcutaneously. The incision was then closed with sterile sutures. Postoperative monitoring was conducted daily to ensure proper wound healing and maintain animal welfare. All mice were housed under standard laboratory conditions (12 h light/dark cycle, 24 °C), with ad libitum access to food and water.

### Nerve conducting velocity analysis

Nerve conduction velocity (NCV) analysis was performed following previously established protocols [20]. Mice were prepared by fully removing fur from the lower back region near the hind limbs. Electrophysiological recordings were obtained using the Nicolet VikingQuest system (Natus Medical, Middleton, WI, USA). For motor NCV measurements, cathodal stimulation was applied at two sites: the sciatic notch and a point 6 mm distal to it. Recording electrodes were placed on the belly of the gastrocnemius muscle, and a ground electrode was positioned on the animal’s back. To minimize bias, all data were analyzed by an independent examiner blinded to the genotype and treatment group. Motor nerve conduction velocity (MNCV) and compound muscle action potential (CMAP) amplitudes were recorded using supramaximal stimulation. For sensory NCV measurements, both the stimulating and recording electrodes were placed on the tail with a 30 mm inter-electrode distance. A ground electrode was again positioned on the animal’s back. Sensory nerve conduction velocity (SNCV) and sensory nerve action potential (SNAP) amplitudes were evaluated by the same blinded examiner.

### Electrophysiological studies

The distal terminal latencies (DTLs), motor nerve conduction velocities (MNCVs), and compound muscle action potentials (CMAPs) of the median, ulnar, radial, peroneal, and tibial nerves were determined using the surface stimulation method. CMAP amplitudes were measured from baseline to negative peak values. Sensory nerve conduction velocities (SNCVs) were obtained from the median, ulnar, and sural nerves using the orthodromic method. Sensory nerve action potentials (SNAPs) were measured from the positive to negative peaks.

### Statistical analysis

All statistical analyses were performed using GraphPad Prism (GraphPad Software, San Diego, CA, USA). For comparisons between two groups, unpaired Student’s *t*-tests were conducted. For comparisons involving three or more groups, data were first tested for normality using the Shapiro–Wilk test. If both normality and homogeneity of variances were satisfied, one-way ANOVA followed by appropriate post hoc tests was performed. If either assumption was violated, the non-parametric Kruskal–Wallis test was used instead.

## Results

### Estradiol restored *Morc2a* p.S87L stability

**Fig. 1:**
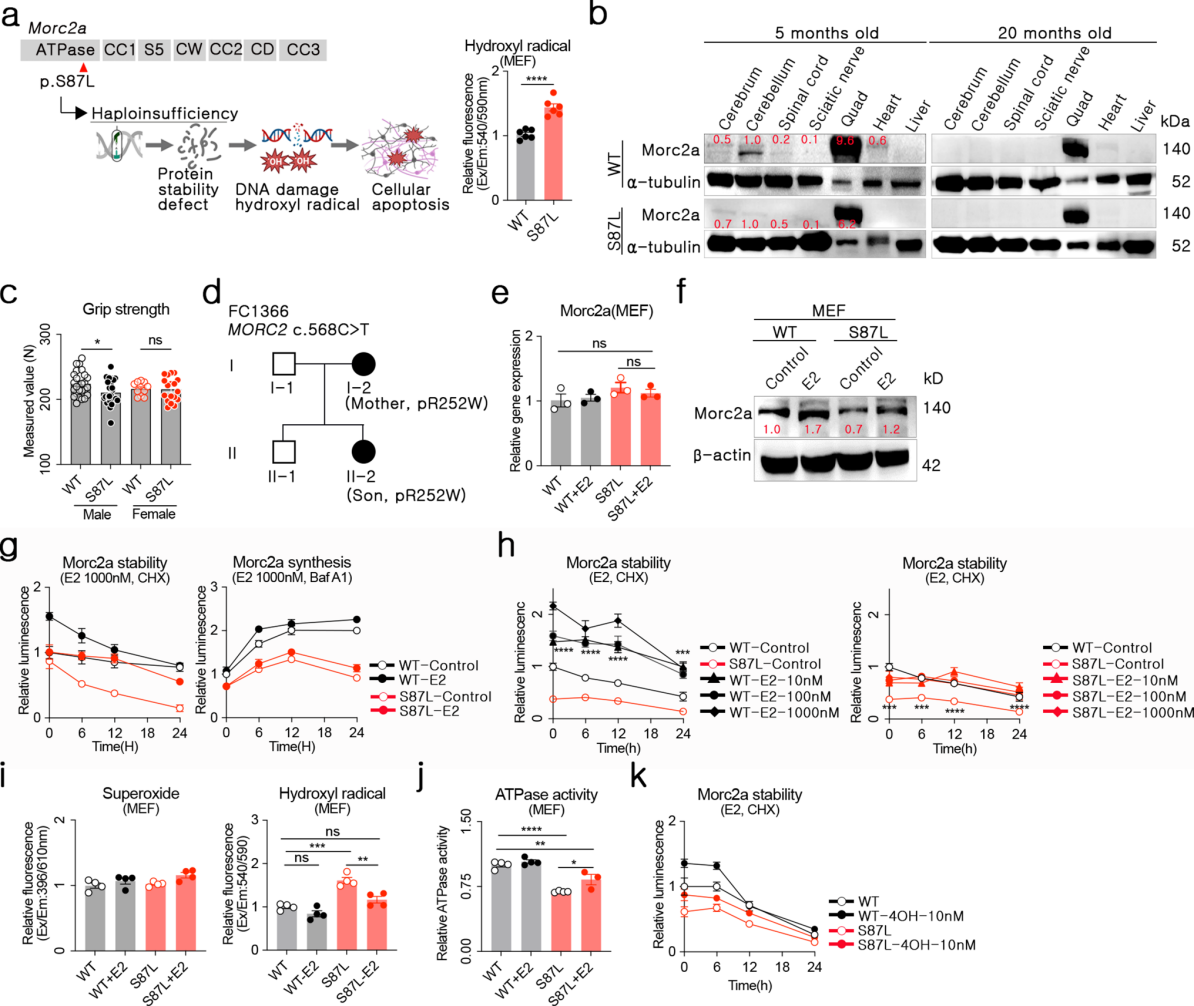
Estradiol (E2)-mediated Microrchidia CW-Type Zinc Finger 2A (*Morc2a*) protein stabilization. **a** Summary of the etiology of Charcot Marie Tooth 2Z (CMT2Z) and cellular hydroxyl radical levels in mouse embryonic fibroblasts (MEFs). **b** Morc2a protein quantification by Western blot in tissues of different organs from wild-type (WT, C57BL/6) and *Morc2a* p.S87L (S87L, C57BL/6.*Morc2a*.^em(S87L)^) mice. **c** Grip strength was measured in mice between 5 to 20 months of age. **d** Pedigree of a human patient showing inheritance of the MORC2 variant from mother to son. Opened rectangle: normal, Closed circle: *MORC2* p.R252W. **e**,** f** *Morc2a* mRNA and protein levels were quantified by qRT-PCR or Western blot after treatment with 1,000 nM estradiol in WT or S87L MEFs. **g**, **h** *Morc2a*/HiBiT or *Morc2a* p.S87L/HiBiT plasmids were transfected into NIH3T3 cells, and estradiol was added 24 h post-transfection. Cycloheximide (CHX) or bafilomycin (BafA1) was used to block protein synthesis or autophagy-mediated degradation, respectively. Morc2a protein levels were measured by HiBiT luminescence assay every 6 h. **i**,** j** Cellular superoxide, hydroxyl radical levels, and ATPase activity were measured after estradiol treatment in WT or S87L MEFs. **k** Morc2a protein was quantified by HiBiT luminescence assay after transfection with WT or S87L *Morc2a*/HiBiT plasmids, followed by estradiol and cycloheximide treatment in NIH3T3 cells. Hereafter, each dot indicates a value from each mouse or cell culture well. Data are presented as the mean ± standard error of the mean (SEM). Statistical analysis was performed using an unpaired Student’s *t*-test, one-way ANOVA or Kruskal–Wallis test. n*s*: not significant, *: *p* < 0.05, **: *p* < 0.01, ***: *p* < 0.001, ****: *p* < 0.0001

The murine *Morc2a* gene is homologous to the human *MORC2* gene. Hydroxyl radicals are key pathological drivers to the neuromuscular pathology associated with *Morc2a* p.S87L and p.R252W variants in CMT2Z (Fig. 1a). Augmentation of *Morc2a* effectively reduced hydroxyl radical accumulation, thereby improving cellular viability [9]. Due to the highly reactive and transient nature of hydroxyl radicals, their direct quantification in tissue remains challenging. Instead, measuring *Morc2a* protein level provided a more reliable approach for identifying tissues most affected by *Morc2a* haploinsufficiency. Muscle weakness emerged in four-month-old *Morc2a* p.S87L mice [19], and progressed to severe demyelination by 15 months of age [26]. Based on these findings, we analyzed protein expression in *Morc2a* p.S87L mice at five and twenty months to investigate early disease onset and post-symptomatic progression in CMT2Z. *Morc2a* protein was predominantly expressed in the cerebellum and *Quadriceps femoris* (Quad). In the cerebellum, *Morc2a* protein was detectable at five months of age but was absent by twenty months. Additionally, Morc2a expression was lower in *Morc2a* p.S87L mice compared to WT controls. In contrast, *Morc2a* protein levels in the Quad remained consistently high regardless of age (Fig. 1b). These findings identified the cerebellum and Quad as key target tissues for further investigation.

Involvement of the Quad was observed among the affected organs resulting from *Morc2a* haploinsufficiency, and oxidative stress-induced mitochondrial damage is implicated in muscle dysfunction [48]. To assess its impact, grip strength analysis was performed in five- to twenty-month-old WT and *Morc2a* p.S87L mice. Male *Morc2a* p.S87L mice exhibited significantly weaker grip strength than WT controls, whereas no genotype-dependent differences were observed in females (Fig. 1c). This pattern suggested the presence of a female-specific protective factor that mitigates muscle weakness. Due to the limited number of patients carrying the *MORC2* p.R252W variant, statistical comparisons of clinical severity between sexes were not feasible. However, case data from patient FC1366 (a mother-son pair) indicated a more severe phenotype in the male patient, with symptom onset at three years of age, compared to his mother, who first reported symptoms at eight years. The son also exhibited greater muscle atrophy and more pronounced proximal weakness, whereas his mother showed predominantly distal muscle involvement. Functional disability scores (CMTNS and FDS) further highlighted more significant impairment in the male patient (Fig. 1d and Supplementary Table 2). Although the small sample size limited generalizability, the mother-son relationship reduced secondary genetic variability, supporting the protective hypothesis associated with the female factor.

Given estrogen’s well-documented neuroprotective effects against oxidative stress and neurodegeneration [29], it emerged as a promising candidate for further investigation. Among estrogen derivatives, estradiol (E2) is the most potent and is FDA-approved for therapeutic applications. Notably, estradiol administration did not increase *Morc2a* mRNA levels (Fig. 1e) but significantly enhanced *Morc2a* protein expression (Fig. 1f), suggesting that its primary mechanism was post-translational rather than transcriptional. To further assess protein stabilization, we utilized a plasmid encoding *Morc2a* tagged with an 11-amino-acid HiBiT sequence under conditions of protein synthesis and autophagy inhibition [9]. Estradiol treatment enhanced *Morc2a* protein expression in both WT and p.S87L variants under cycloheximide-induced protein synthesis inhibition, whereas no changes were observed under autophagy inhibition via BafA1 (Fig. 1g). These results suggest that estradiol stabilized *Morc2a* protein by preventing degradation rather than affecting synthesis, thereby counteracting autophagy. Notably, even a low dose (10 nM) of estradiol increased *Morc2a* p.S87L protein stability by approximately 85%, restoring its levels to those observed in WT (Fig. 1h).

Building on these observations, we hypothesized that estradiol alleviates *Morc2a* haploinsufficiency by attenuating hydroxyl radical-mediated apoptosis and restoring ATPase activity. Indeed, estradiol treatment significantly reduced hydroxyl radical levels while increasing ATPase activity (Fig. 1i and 1j), mirroring the effects observed with *Morc2a* gene augmentation therapy [9]. Furthermore, estradiol more effectively normalized hydroxyl radical levels in *Morc2a* p.S87L MEFs than in WT cells, fully restoring them to WT levels. Given the clinical relevance of estrogenic compound, we also examined the applicability of selective estrogen receptor modulators (SERMs), such as tamoxifen. Similar to estradiol, tamoxifen treatment increased Morc2a protein stability in both WT and p.S87L variants (Fig. 1k), further supporting the therapeutic potential of estrogen-based compounds. To maintain coherence in our research, subsequent studies were conducted based on estradiol.

### *Morc2a* function was restored by estradiol independently of specific estrogen receptors

**Fig. 2:**
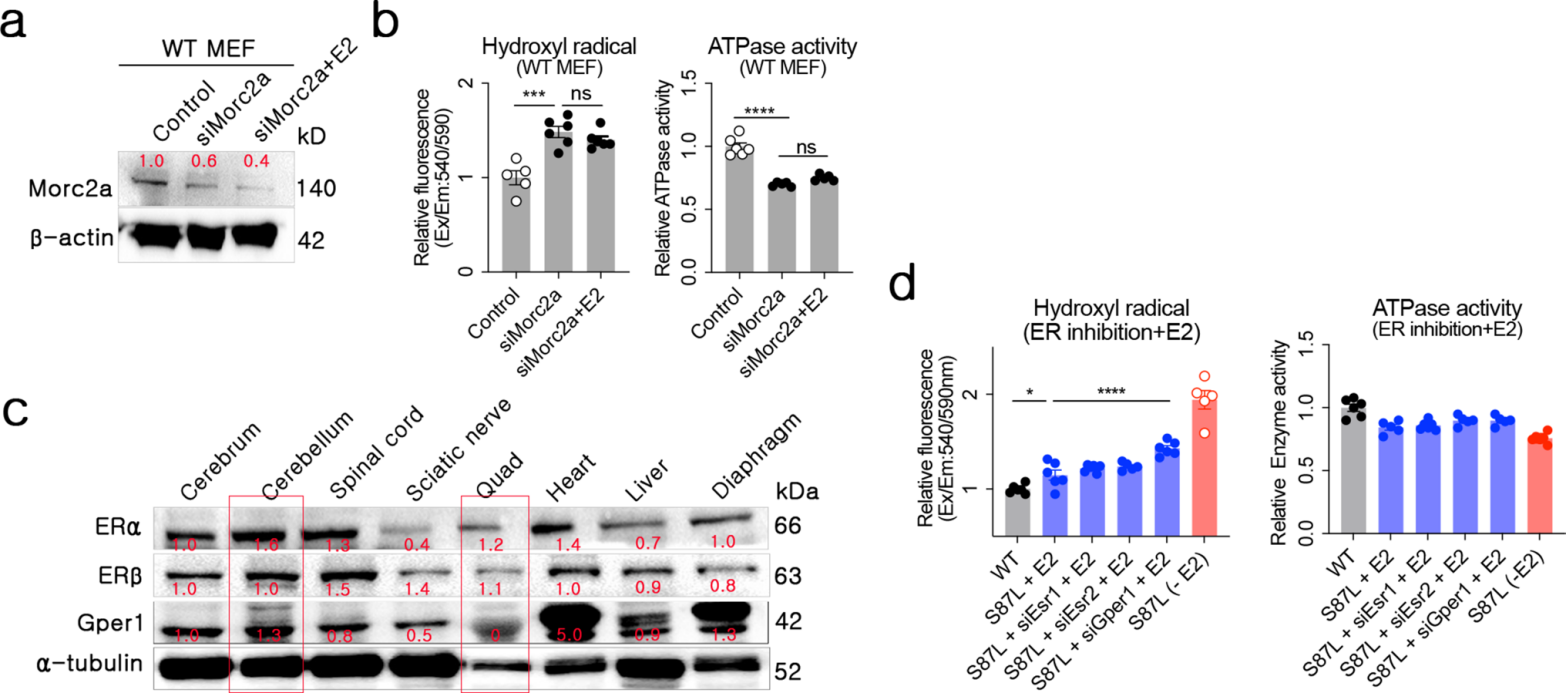
Morc2a stabilization by estradiol through interaction with ER alpha (ERα), ER beta (ERβ), and G protein-coupled estrogen receptor 1 (Gper1). **a** Morc2a protein levels were quantified after si*Morc2a* and estradiol treatment in WT MEFs. **b** Cellular hydroxyl radical levels and ATPase activity were measured after si*Morc2a* and estradiol treatment. **c** Quantification of estrogen receptors (ERs) in tissues from various organ of WT mice. **d** Hydroxyl radical levels and ATPase activity were measured following knockdown of each ER using siRNA under estradiol treatment

To assess estradiol as a therapeutic agent for CMT2Z, we needed to investigate its potential activity in the cerebellum and Quad. Estrogen receptors (ERs) regulate transcription of various genes [22] and promote protein stability by inhibiting degradation through the ubiquitin–proteasome system [18]. Given the potential for estradiol to influence cellular viability independently of *Morc2a*, we analyzed its therapeutic effects under *Morc2a*-inhibited conditions. In *Morc2a*-inhibited MEFs, estradiol treatment did not increase *Morc2a* protein levels (Fig. 2a). Furthermore, high cellular hydroxyl radical concentration and low ATPase activity were observed regardless of the presence of estradiol under Morc2a inhibition (Fig. 2b). These findings demonstrated that estradiol-mediated reductions in hydroxyl radical levels and the restoration of ATPase activity in *Morc2a* p.S87L cells depended strictly on functional *Morc2a*.

Estradiol functions primarily through binding to ERs, which include ER alpha (ERα), ER beta (ERβ), and G protein-coupled estrogen receptor 1 (Gper1). These receptors show distinct, tissue-specific expression patterns [8]. ERα and ERβ were expressed in most mouse organs, whereas Gper1 showed a distinct expression pattern between neuronal and non-neuronal system. Particularly in the primary affected organs in CMT2Z, all three ER subtypes were expressed in the cerebellum, whereas Quad predominantly expressed ERα and ERβ with relatively low levels of Gper1 (Fig. 2c). To determine the specific ER subtype responsible for estradiol's effects in *Morc2a* haploinsufficiency, we used siRNA to silence ERα, ERβ, or Gper1 in *Morc2a* p.S87L cells followed by estradiol treatment. Estradiol effectively reduced hydroxyl radical levels and restored ATPase activity even when each ER subtype was individually silenced (Fig. 2d). This suggested that estradiol-mediated stabilization of *Morc2a* protein did not rely on any single ER subtype. However, therapeutic effects were least pronounced upon Gper1 inhibition, implying that estradiol might be more effective in tissues with higher Gper1 expression. Nevertheless, estradiol still significantly reduced hydroxyl radical levels by approximately 25% compared to untreated *Morc2a* p.S87L cells, even under Gper1-silenced conditions (Fig. 2d). This indicated that estradiol could provide therapeutic benefits even in tissues with lower Gper1 expression, such as Quad. Taken together, these data highlight estradiol as a promising therapeutic candidate for both cerebellum and Quad, two key tissues affected in CMT2Z.

### Estradiol restores *Morc2a* stability and reduces oxidative mitochondrial aggregation

**Fig. 3:**
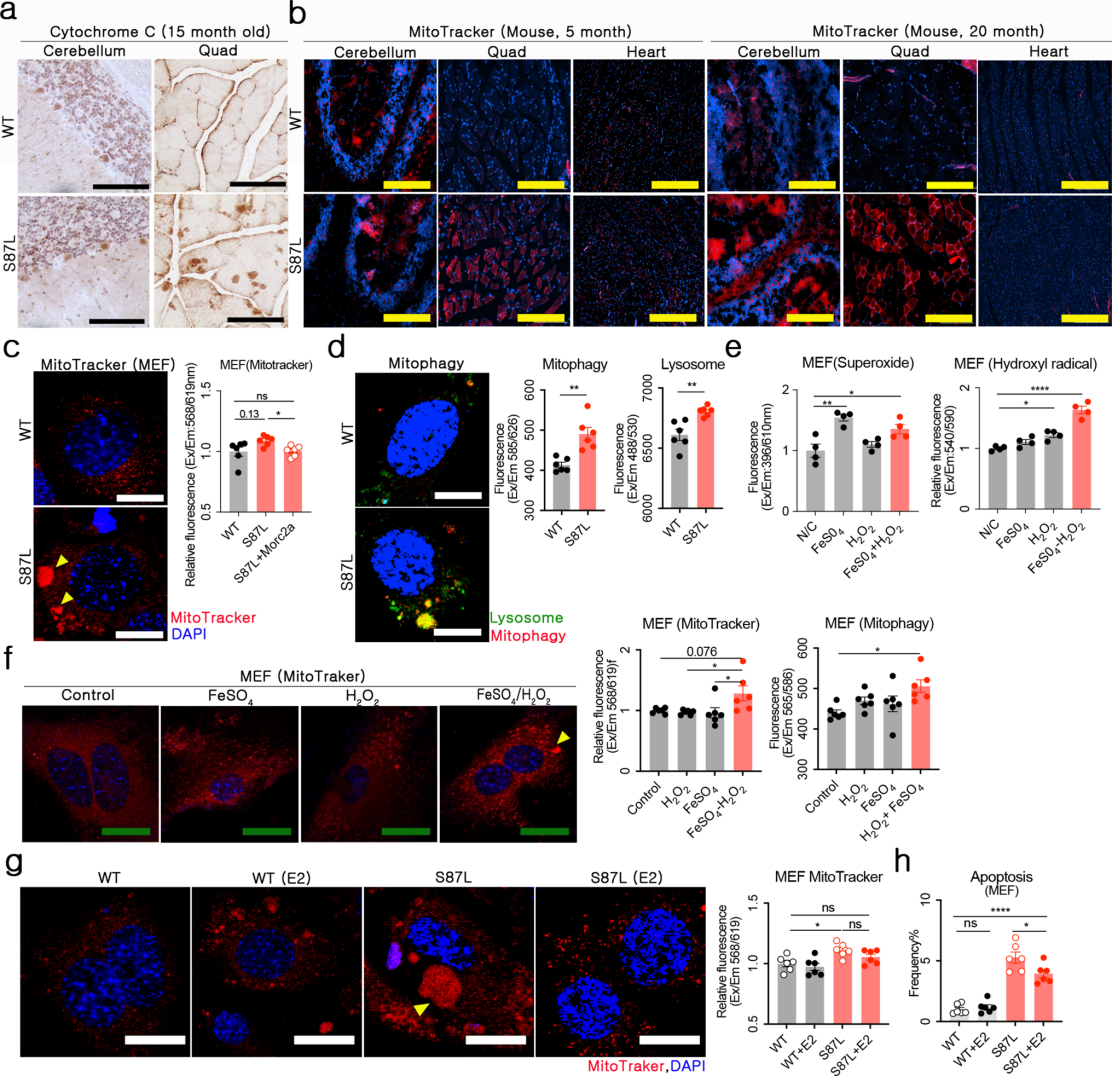
Mitochondrial aggregation in the cerebellum and *Quadriceps femoris* (Quad) under *Morc2a* haploinsufficiency. **a** Detection of cytochrome c by immunohistochemistry in the cerebellum and Quad. (black scale bar = 100 μm) **b** Visualization of MitoTracker signals in the cerebellum, Quad, and heart from 5- and 20-month-old mice (yellow scale bar = 100 μm). **c** Representative confocal microscopy images of MEFs after MitoTracker staining. White triangles indicate aggregated mitochondria. (white scale bar = 25 μm) **d** Immunofluorescence for mitophagy and lysosomes in WT and S87L MEFs (white scale bar = 25 μm). **e** Quantification of cellular superoxide and hydroxyl radicals after Fe₂SO₄, H₂O₂, or Fe₂SO₄-H₂O₂ treatment. **f** MitoTracker signal detection after Fe₂SO₄, H₂O₂, or Fe₂SO₄-H₂O₂ treatment in WT MEFs (green scale bar = 50 μm). **g** Representative confocal images showing mitochondrial aggregation after estradiol treatment in WT or S87L MEFs (white scale bar = 25 μm). **k** The frequency of apoptotic cells was calculated after estradiol treatment in WT or S87L MEFs

Intrinsic apoptosis disrupts mitochondrial membrane integrity, releasing cytochrome c into the cytosol [4]. Aggregated cytochrome c and cleaved caspase 3 were prominent in the Purkinje cells of the cerebellum and central regions of Quad muscle fibers in Morc2a p.S87L mice (Fig. 3a and Supplementary Fig. 1). To further evaluate mitochondrial integrity, MitoTracker staining was performed to examine changes in mitochondrial membrane potential, morphology, distribution, and abundance [7]. In *Morc2a* p.S87L mice, MitoTracker signal intensity was significantly elevated in the cerebellum and Quad at five and twenty months old (Fig. 3b and Supplementary Fig. 2). An intensified MitoTracker signal can result from high membrane potential or mitochondrial fusion [16, 41]. However, in *Morc2a* p.S87L mice, the primary cause of this elevated signal was mitochondrial aggregation, as *Morc2a* supplementation markedly reduced MitoTracker intensity (Fig. 3c). These findings indicated that *Morc2a* haploinsufficiency promotes mitochondrial aggregation. This phenomenon was a hallmark of apoptosis and precedes cytochrome c release [13]. In *Morc2a* p.S87L mouse embryonic fibroblasts (MEFs), mitochondrial aggregation and mitophagy were detected at the same subcellular locations, suggesting a direct link between mitochondrial aggregation and mitochondrial death (Fig. 3d). To determine whether hydroxyl radicals directly trigger mitochondrial aggregation, we experimentally induced hydroxyl radical formation by treating cells with FeSO_4_ and H_2_O_2_ [2]. Although FeSO_4_ alone elevated superoxide levels, significant hydroxyl radical production occurred only when FeSO_4_ and H_2_O_2_ were co-administered (Fig. 3e). Under these controlled ROS-inducing conditions, only hydroxyl radicals, rather than superoxide or H_2_O_2_, induced mitochondrial aggregation and intensified MitoTracker signal in WT MEFs (Fig. 3f). These findings demonstrated that hydroxyl radical-mediated mitochondrial aggregation plays a central role in apoptotic signaling in *Morc2a* p.S87L MEFs, cerebellum, and Quad. To evaluate whether estradiol could counteract the mitochondrial defects associated with *Morc2a* haploinsufficiency, we examined its effect on mitochondrial aggregation and apoptosis in *Morc2a* p.S87L MEFs. As expected, estradiol treatment markedly reduced both mitochondrial aggregation and cellular apoptosis (Fig. 3g and 3h). These findings demonstrate that estradiol enhances *Morc2a* protein stability, alleviates hydroxyl radical-induced apoptotic signaling, and preserves mitochondrial homeostasis in Morc2a-deficient cells.

### *MORC2* stabilization and functional recovery recapitulated in human fibroblast and motor neuron

**Fig. 4:**
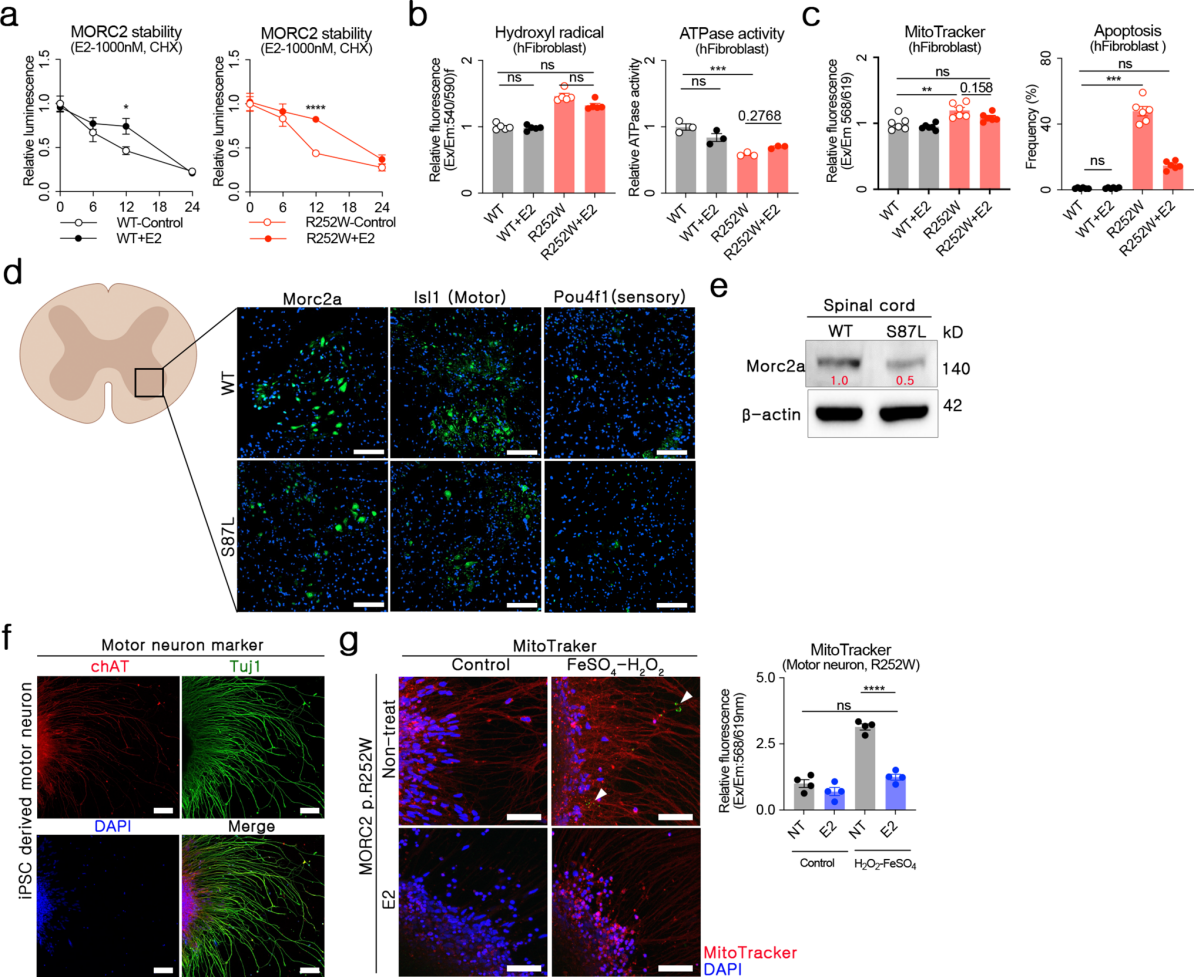
Enhanced viability of *MORC2* variant motor neurons by estradiol treatment. **a** MORC2 protein levels were quantified using a HiBiT luminescence assay after transfection with *MORC2*/HiBiT or *MORC2* p.R252W/HiBiT plasmids. Cycloheximide was used to inhibit protein synthesis. **b**, **c** Cellular hydroxyl radical levels, ATPase activity, MitoTracker signals, and the frequency of apoptotic cells were measured in human WT or *MORC2* p.R252W fibroblasts after treatment with 1000 nM estradiol. **d, e** Detection and quantification of Morc2a in the anterior horn of the spinal cord from 5-month-old mice. ISL LIM Homobox1 (Isl1, motor neuron), Pit-oct-unc-4 class factor 1 (Pou4f1, sensory neuron) (white scale bar = 100 μm) **f** Induced pluripotent stem cells (iPSC) were generated from patient fibroblasts carrying the *MORC2* p.R252W mutation. After differentiating iPSCs into motor neurons, the differentiation was validated using choline acetyltransferase (ChAT) and neuron-specific class III beta-tubulin (Tuj1) markers. **g** Representative image and graph of aggregation of mitochondria with estradiol treatment or high hydroxyl radical condition in MORC2 p.R252W derived motor neurons. Analyses were primarily conducted in the region associated with axon generation. (white scale bar = 100 μm)

Validating the therapeutic efficacy of estradiol observed in mouse models using human *MORC2* variants was critical for advancing treatment strategies. Because *Morc2a* haploinsufficiency and hydroxyl radical-mediated cellular apoptosis observed in *Morc2a* p.S87L mouse cells were similarly present in human *MORC2* p.R252W variant cells [9], we hypothesized that estradiol would exhibit comparable therapeutic effects in human cells. Indeed, estradiol treatment enhanced protein stability of both wild-type (WT) and *MORC2* p.R252W proteins (Fig. 4a), reduced hydroxyl radical levels, and restored ATPase activity, thereby mitigating MORC2 haploinsufficiency (Fig. 4b). Consistently, estradiol decreased MitoTracker signal intensity in *MORC2* p.R252W cells, indicative of reduced mitochondrial aggregation and apoptosis (Fig. 4c). While estradiol treatment induced minimal changes in hydroxyl radical concentrations, ATPase activity, mitochondrial aggregation, or apoptosis in WT fibroblasts, it significantly improved these parameters in *MORC2* p.R252W fibroblasts, aligning with observations in *Morc2a* p.S87L mouse cells (Fig. 1i, 1j, and 4c).

Although fibroblast-based assays demonstrated the potential of estradiol to rescue MORC2 haploinsufficiency, the primary pathological site in CMT2Z is neuronal tissue. Our previous study reported increased cellular apoptosis in the spinal cords of *Morc2a* p.S87L mice [9], although it did not specify which cell types were predominantly affected by the reduction in *Morc2a* expression. In the present study, we confirmed that *Morc2a* is expressed in the motor neuron region of the anterior horn of the mouse spinal cord (Fig. 4d), and its protein levels are reduced in the spinal cords of *Morc2a* p.S87L mice (Fig. 4e). These findings provide a strong rationale for investigating the therapeutic effects of estradiol specifically in motor neurons. We therefore generated induced pluripotent stem cells (iPSCs) from fibroblasts of *MORC2* p.R252W patients and differentiated them into motor. Following validation using motor neuron-specific markers choline acetyltransferase (chAT) and neuron-specific class III beta-tubulin (Tuj1) (Fig. 4f) [33], we assessed neuronal injury induced by hydroxyl radicals and evaluated the therapeutic effects of estradiol treatment (Fig. 4g). Estradiol significantly reduced MitoTracker signal intensity in *MORC2* p.R252W motor neurons. Importantly, mitochondrial aggregation was elevated considerably in motor neurons exposed to FeSO_4_-H_2_O_2_, and mitochondrial changes were substantially mitigated by estradiol treatment. Apoptotic signals were occasionally observed in FeSO_4_-H_2_O_2_-treated motor neurons (white triangle, Fig. 4g), but their frequency was too low for statistical analysis (Fig. 4g and Supplementary Fig. 3).

### Estradiol treatment improved muscular function and reduced cellular apoptosis in aged *Morc2a* p.S87L mice

**Fig. 5:**
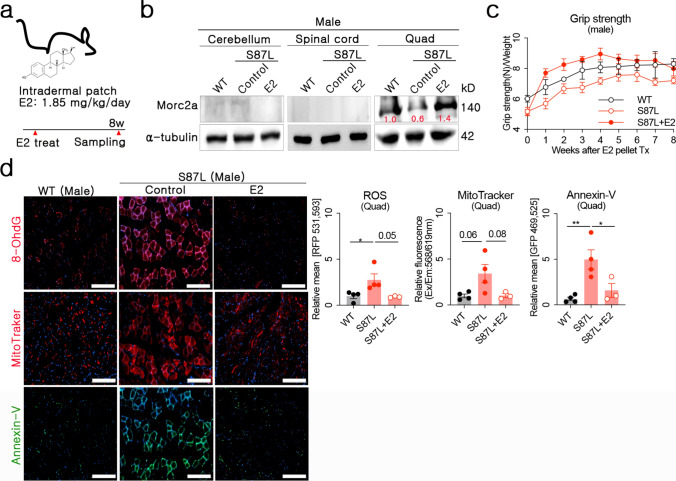
Therapeutic effect of estradiol treatment on Quad. **a** Schematic overview of the estradiol treatment. Five milligrams of estradiol or placebo pellets were implanted subcutaneously into the dorsal trunk. Grip strength was measured weekly, and organ samples were collected eight weeks after treatment initiation. **b** Morc2a protein quantification in the cerebellum, spinal cord and Quad after estradiol treatment. **c** Grip strength was measured weekly (n = 6 per group). **d** Representative immunofluorescence images and quantification of 8-OHdG, MitoTracker, and Annexin-V staining in the Quad eight weeks after estradiol treatment. (white scale bar = 200 μm)

Cell-based analyses in both murine and human models consistently demonstrated that estradiol effectively rescued *Morc2a* haploinsufficiency and mitigates apoptosis. Given that ER expression was confirmed across various murine tissues, we hypothesized that estradiol would also exert therapeutic effects in vivo. Pellet implantation was selected for estradiol delivery, enabling sustained release and prolonged therapeutic efficacy. *Morc2a* p.S87L mice exhibit distinct clinical phenotypes starting between 6–9 months of age [19]. To evaluate the therapeutic potential of estradiol after disease onset, we used 18 month-old mice as a late-stage disease model. Human hormone replacement therapy typically employs estradiol doses ranging from 1–2 mg/kg/day [30], and mouse prostate cancer studies have used a dosage of 5 mg/kg/week [27]. Guided by these reports, we subcutaneously implanted 5 mg pellets of 17β-estradiol into the dorsal trunk. This ensured a sustained release rate of 1.85 mg/kg/day for 90 days (Fig. 5a).

As estradiol effects were anticipated to be more pronounced in male mice, we initially focused our analyses on male *Morc2a* p.S87L mice. Protein quantification confirmed that estradiol administration successfully alleviated *Morc2a* haploinsufficiency, significantly enhancing *Morc2a* protein levels in Quad. Notably, *Morc2a* expression remained undetectable in the cerebellum and spinal cord following treatment (Fig. 5b), consistent with estradiol's proposed role in protein stabilization rather than transcriptional induction. Given that estradiol’s therapeutic effects rely on existing *Morc2a* expression (Fig. 2b), we anticipated functional improvements primarily in skeletal muscle rather than the cerebellum. Indeed, estradiol significantly improved muscular function within one week of administration, with effects persisting for more than eight weeks (Fig. 5c). Histopathological evaluation further highlighted substantial improvements in muscle integrity following estradiol treatment. *Morc2a* p.S87L mice exhibited cerebellar ataxia, characterized by movement incoordination and gait abnormalities [9], but this phenotype was less associated with a loss of muscle strength [23]. Therefore, the recovery of grip strength was interpreted as a therapeutic effect on skeletal muscle. Prior to estradiol administration, male *Morc2a* p.S87L mice exhibited elevated reactive oxygen species (ROS), increased MitoTracker signal intensity, and enhanced cellular apoptosis compared to WT mice. Estradiol treatment effectively reversed these pathological features, restoring them to WT levels (Fig. 5d). These findings underscore estradiol’s potent protective effects on muscular pathology in aged male *Morc2a* p.S87L mice. Our subsequent interest was evaluating the effects of estradiol treatment in female *Morc2a* p.S87L mice. Estradiol administration in 18-month-old female *Morc2a* p.S87L mice did not improve grip strength in the Quad (Supplementary Fig. 4a). Pathological assessment revealed an unexpected increase in 8-OHdG levels within Quad tissues following estradiol treatment. Although reductions in MitoTracker signals and apoptotic cells were observed, these changes did not reach statistical significance (Supplementary Fig. 4b). These results observed in female *Morc2a* p.S87L mice demonstrated both beneficial and adverse effects. In summary, the therapeutic efficacy of estradiol was clearly evident in male *Morc2a* p.S87L mice, particularly in alleviating muscular pathology.

### Estradiol ameliorates peripheral neuropathy in 20-month-old *Morc2a* p.S87L mice

**Fig. 6:**
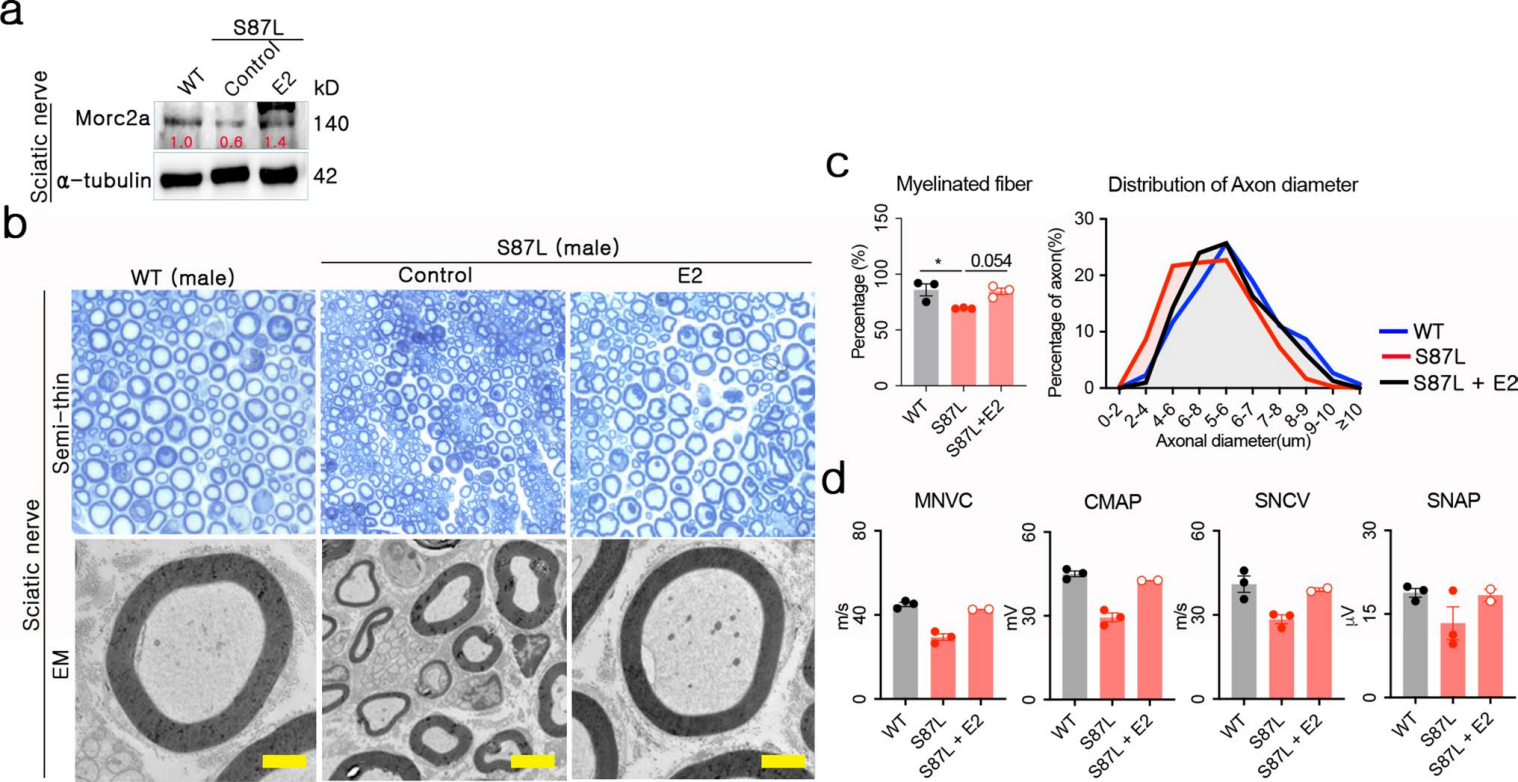
Estradiol enhanced sciatic nerve integrity and function. **a** Quantification of Morc2a in the Quad 8 weeks after estradiol treatment. **b** Representative semi-thin sections (magnification × 100) and ultrathin section observed by electron microscopy. Yellow scale bar = 2 μm. **c** The frequency of myelinated neuron fibers, axonal diameter were calculated from three different regions (200 × 200 μm) in each mouse, and average score was subjected to analysis. **d** Nerve conduction velocity analysis of motor and sensory neurons in the sciatic nerve after estradiol treatment. MNCV = motor nerve conducting velocity; CMAP = compound muscle action potential; SNVC = sensory nerve conduction velocity; SNAP = sensory nerve action potential; SNVC = sensory nerve conduction velocity

Unlike the central nervous system, axonal neuropathy in the mammalian peripheral nervous system can regenerate following therapeutic intervention [14]. It is widely accepted that peripheral neuropathies commonly result in muscular degeneration subsequent to nerve damage. However, some studies have suggested that neuronal and muscular pathologies may occur independently in neuropathies associated with *MORC2* variants [15]. Given these complexities, we investigated whether estradiol-induced muscle recovery coincided with direct therapeutic improvements in peripheral nerve pathology. Clinically, diagnosis of CMT2 involves evaluating muscular weakness and foot deformities, corroborated by electrophysiological assessments [3]. As muscular function had previously been assessed in the Quad, we analyzed therapeutic efficacy using the sciatic nerve, a representative peripheral nerve. *Morc2a* protein was marginally detectable in the sciatic nerves across all genotypes, with its expression being enhanced by estradiol treatment (Fig. 6a). This upregulation of *Morc2a* was anticipated to exert therapeutic effects on the sciatic nerve, contributing to both structural and functional improvement in CMT2Z. Furthermore, we expected that mitigating hydroxyl radical levels in the surrounding skeletal muscles could potentiate the therapeutic impact on the sciatic nerve.

We performed electron microscopy analyses on sciatic nerves to confirm the potential reversal of peripheral neuropathy by estradiol. Before treatment, male *Morc2a* p.S87L mice displayed typical features of axonal neuropathy, including decreased number of myelinated fibers and reduced axonal diameters. Remarkably, estradiol treatment effectively restored sciatic nerve morphology to levels comparable with WT controls (Fig. 6b). Quantitative analysis further substantiated these findings, revealing that estradiol significantly increased both the proportion of large-myelinated fibers and axonal diameters in *Morc2a* p.S87L mice (Fig. 6b and 6c). These results strongly indicate a regenerative and restorative role of estradiol in peripheral nerve integrity.

Histological evidence of sciatic nerve recovery prompted an investigation into whether functional improvements accompanied structural restoration. To evaluate sensory and motor nerve functionality, we performed nerve conduction velocity (NCV) analysis, a key diagnostic measure for axonal neuropathy in CMT2Z [3]. Although unexpected death of aged mice precluded statistical analysis of electrophysiological parameters, available data indicated that estradiol treatment restored both sensory and motor nerve conduction velocity, as well as action potential amplitudes, in *Morc2a* p.S87L mice to WT levels (Fig. 6d). These findings strongly suggested that estradiol reversed neurodegeneration and restored functional capacity in symptomatic mice. In summary, estradiol mediated CMT2Z treatment effectively reversed sciatic nerve degeneration, demonstrating structural restoration alongside functional recovery. Notably, these therapeutic effects, characterized by regeneration rather than prevention, represent distinct findings that were not observed in our previous AAV-mediated gene therapy study [9].

## Discussion

This study demonstrates a cost-effective and readily applicable therapeutic strategy for CMT2Z. Some *Morc2a* mutations lead to haploinsufficiency caused by protein instability, which results in neuromuscular disease through cellular apoptosis triggered by low ATPase activity and elevated hydroxyl radicals. While our previous study achieved therapeutic effects using a hydroxyl radical scavenger, a more fundamental treatment approach involves restoring *Morc2a* to normalize all associated cellular functions, given the additional pathogenic role of reduced ATPase activity. Estradiol increased the resistance of *Morc2a* protein to autophagic degradation, restoring *Morc2a* protein levels and exerting therapeutic effects in CMT2Z. Notably, this mechanism was also reproduced in human *MORC2* p.R252W mutations. Our study demonstrates that estradiol administration improves peripheral nerve and muscle damage in symptomatic CMT2Z animal models and restores function to near-normal levels.

Hydroxyl radicals have been implicated in neuropathic degeneration; however, the underlying mechanisms remain poorly understood. Our previous study identified *Morc2a* haploinsufficiency as a key pathogenic factor driving hydroxyl radical–mediated DNA damage and axonal neuropathy in [9]. Notably, the primary sites of degeneration in CMT2Z—the cerebellum, spinal cord, sciatic nerve, and distal skeletal muscles—correspond to regions with high endogenous *Morc2a* expression, supporting the hypothesis that stabilizing *Morc2a* may reduce hydroxyl radical accumulation and thereby mitigate mitochondrial dysfunction and axonal loss. The present study builds on these findings by demonstrating that estradiol confers a therapeutic benefit by attenuating hydroxyl radical–induced neuropathy in the CMT2Z model. Previous work in breast cancer cells has shown that estradiol inhibits lysosomal degradation of *MORC2* through the GPER–PRKACA–chaperone-mediated autophagy pathway[46]. While that study focused on *MORC2* overexpression and tumor progression, our results emphasize estradiol’s capacity to stabilize *Morc2a* protein under haploinsufficient conditions. This context-dependent regulatory mechanism provides a plausible basis for the observed neuroprotective effects of estradiol in CMT2Z. Despite these promising findings, the precise molecular cascade by which *Morc2a* deficiency leads to increased hydroxyl radical production remains to be elucidated. Likewise, the mechanisms linking mitochondrial aggregation to impaired ATPase activity are not yet fully understood. These unresolved questions underscore the need for further investigation to define the mechanistic interplay between *Morc2a*, hydroxyl radicals, and their contributions to neuronal aggregation and apoptosis.

CMT2Z presents with severe clinical manifestations, yet no widely applicable and affordable treatment has been developed. Therefore, targeting *Morc2a* stabilization with estradiol appears to be a potential strategy to address the key pathophysiological features of high hydroxyl radical levels and low ATPase activity. Given that synthetic estrogens have been FDA-approved as early as 1941 and are widely used for conditions such as menopausal symptom relief, osteoporosis prevention, and ovarian failure [17], estradiol could be repurposed as a viable treatment for CMT2Z. However, estrogen hormone replacement therapy has been associated with adverse effects, including an increased risk of breast cancer and thromboembolism [42]. In ER-positive breast cancer, estrogen-mediated MORC2 phosphorylation via the GPER1 pathway inhibits chaperone-mediated autophagy, leading to increased MORC2 protein expression [46] and promoting cancer cell proliferation and metastasis [21]. While estradiol-induced MORC2 upregulation poses a potential risk in breast cancer [50], liver cancer has also been observed in Morc2a-deficient *Morc2a* p.S87L mice. This suggests that MORC2 homeostasis loss plays a more critical role in tumorigenesis [9]. Furthermore, as a hydrophobic molecule, estrogen can cross the blood–brain barrier and reach the spinal cord and peripheral nerves through the bloodstream, making it advantageous for preventing MORC2 haploinsufficiency-mediated neuropathy [39]. Based on these findings, we propose that MORC2 upregulation by estradiol could serve as a therapeutic strategy for *Morc2a* haploinsufficiency-driven CMT2Z.

Numerous neurological disorders exhibit sex-based differences in disease severity. For example, Parkinson’s disease is more prevalent and progresses more rapidly in males, whereas estrogen has been shown to exert neuroprotective effects in females by attenuating oxidative stress and inflammation in dopaminergic neurons. Notably, the incidence of Parkinson’s disease increases in postmenopausal women, aligning with the loss of estrogen-mediated protection [28, 40]. Similarly, in amyotrophic lateral sclerosis (ALS), estrogen contributes to mitochondrial stabilization and is associated with lower disease incidence and slower progression in females [5]. In the present study, we observed a more severe neuromuscular phenotype in male *Morc2a* p.S87L mice than in females. This observation is consistent with limited clinical evidence suggesting potential sex-based differences in CMT2Z, although the underlying mechanisms remain poorly understood. Notably, estradiol administration significantly reduced apoptosis in the Quad of male mice, suggesting that estrogen-mediated neuroprotection may contribute, at least in part, to sex-related differences in disease severity. We hypothesize that this protective effect may be achieved through the mitigation of ROS–induced mitochondrial damage. However, further studies are required to delineate the mechanistic interplay among estrogen, *Morc2a*, hydroxyl radicals, and mitochondrial dysfunction.

Next, a critical consideration is whether estradiol administration is feasible for male CMT2Z patients. Although estradiol has been investigated for the treatment of prostate cancer through testosterone suppression, cardiovascular and thromboembolic risks remain concerns [36]. Given these potential risks, further studies are necessary to develop low-dose parenteral administration strategies for estradiol or selective estrogen receptor modulators (SERMs) to maximize therapeutic effects while minimizing adverse events. In this study, tamoxifen, a representative SERM, was also found to enhance *Morc2a* protein stability, suggesting that both estrogen and ER-targeted therapies could serve as potential treatments. The long-term outcomes of estradiol administration—including the persistence of therapeutic efficacy and potential adverse effects on the cardiovascular, central nervous, metabolic, or oncogenic systems—were not evaluated in this study and should be addressed in future investigations. In the present study, a 90-day slow-release estradiol pellet was used to ensure sustained systemic exposure in the animal model. However, therapeutic efficacy was assessed at 8 weeks of age; therefore, the long-term safety and durability of estradiol treatment in CMT2Z remain to be elucidated. Given the availability of numerous FDA-approved estrogen analogs and SERMs with established efficacy and safety profiles [32], patient treatment can be rapidly implemented once optimal therapeutic candidates and regimens are identified. Furthermore, while estradiol administration in female disease models exacerbated certain disease markers, highlighting the need for additional studies to investigate the underlying mechanisms and determine appropriate therapeutic dosage ranges, the use of estradiol-based therapies for female CMT2Z patients warrants careful consideration and further exploration.

Peripheral neuropathy is typically diagnosed after clinical symptoms manifest, underscoring the need for therapies that restore function to damaged tissues rather than focusing solely on prevention. Unlike the central nervous system, which exhibits limited regenerative capacity [45], this study provides compelling evidence that restoring *Morc2a* expression promotes significant regeneration and functional recovery in affected peripheral nerves and muscles. The *Morc2a* stabilization strategy using estradiol is predicated on the presence of *Morc2a* protein expression. Therefore, therapeutic effects may be limited in tissues where gene expression is lost after disease onset. In such cases, AAV-based gene therapy to induce *Morc2a* expression in affected tissues may offer a more direct treatment approach [9]. Nevertheless, considering the absence of an FDA-approved gene therapy currently available for CMT2Z, estradiol-based interventions are a practical and widely accessible alternative. Furthermore, personalized treatment strategies tailored to disease stage and symptoms can be developed by analyzing the affected organs and the corresponding MORC2 expression levels in each.

## Conclusions

This study identifies hydroxyl radicals as contributors to neuropathy through mitochondrial aggregation and apoptosis and demonstrates that estradiol restores hydroxyl radical homeostasis through the stabilization of *Morc2a*. Consequently, estradiol directly addresses *Morc2a* haploinsufficiency-driven CMT2Z and can be rapidly repurposed through either existing estradiol formulations or optimized SERMs. Additionally, these findings may apply to other hydroxyl radical-mediated neuromuscular diseases yet to be fully characterized. Rare diseases caused by genetic mutations have historically been overlooked in drug development. Rather than focusing solely on orphan drug development, identifying common disease mechanisms and applying broad-spectrum therapeutics is a more pragmatic approach. This study offers novel insights into developing treatment strategies for related neuromuscular disorders.

## Supplementary Information

Below is the link to the electronic supplementary material.

Supplementary file1 (DOCX 4312 KB)
