# Supplementary material for: Estradiol rescues male hydroxyl radical-mediated Charcot-Marie-Tooth 2Z by Morc2a stabilization through autophagy inhibition in a murine model
Source: Acta Neuropathol. 2025 Aug 4;150(1):13. doi: 10.1007/s00401-025-02922-2 (PMC12321673; doi:10.1007/s00401-025-02922-2)
Supplement: Supplementary file 1 — Supplementary file1 (DOCX 4312 KB) [file 401_2025_2922_MOESM1_ESM.docx]

**Full title**: Estradiol rescues male hydroxyl radical-mediated Charcot-Marie-Tooth 2Z by Morc2a stabilization through autophagy inhibition in a murine model

**Supplementary information**

**Supplementary Tables 1 - 2**

1. Antibody information and siRNA sequences used in this study
2. Clinical characteristics in individuals with *MORC2* mutation

**Supplementary Figures 1 - 4**

1. Detection of cleaved Caspase-3 by immunohistochemistry in the cerebellum and Quad.
2. Quantification and representative images of MitoTracker staining in various organs from WT and p.S87L mice at 5 or 20 months of age.
3. Highly magnified image of MitoTracker signals under estradiol treatment or high hydroxyl radical conditions in motor neurons derived from MORC2 p.R252W.
4. Effect of estradiol treatment on the Quad of female *Morc2a* p.S87L mice.


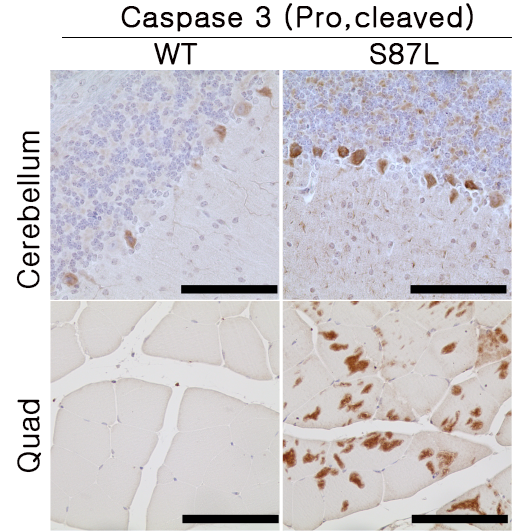


**Fig. S1**. Detection of cleaved Caspase-3 by immunohistochemistry in the cerebellum and Quad. (black scale bar = 100 μm)


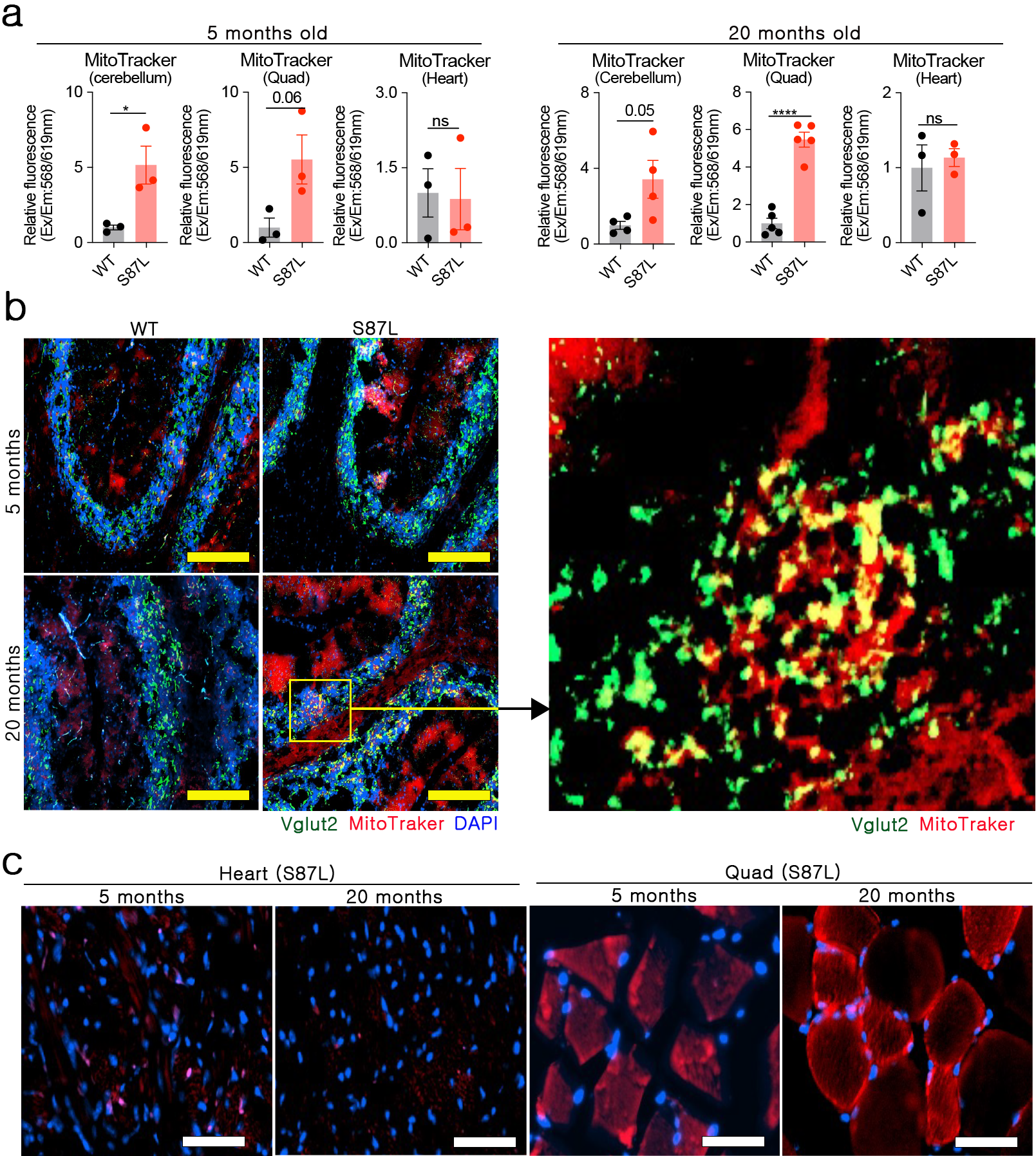


**Fig. S2**. Quantification and representative images of MitoTracker staining in various organs from WT and p.S87L mice at 5 or 20 months of age. **a** Quantitative analysis of MitoTracker signal intensity in multiple organs. **b** MitoTracker localization in the cerebellum of WT and p.S87L mice. MitoTracker signals were predominantly detected in the molecular layer and white matter. Notably, vGlut2-positive neurons co-localizing with MitoTracker were also observed in the granule cell layer. **c** MitoTracker signal in the heart and quadriceps muscle. MitoTracker expression was clearly visualized in individual muscle fibers of the quadriceps. Scale bar; yellow = 200 μm, white =50 μm


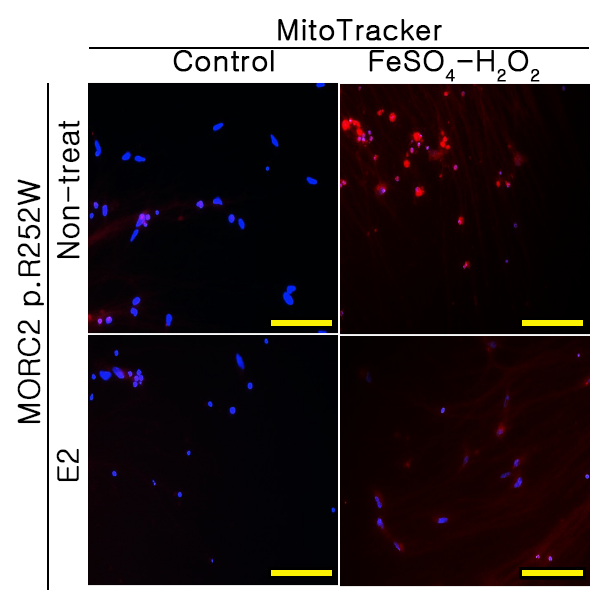


**Fig. S3**. Highly magnified image of MitoTracker signals under estradiol treatment or high hydroxyl radical conditions in motor neurons derived from MORC2 p.R252W. (white scale bar = 100 μm)


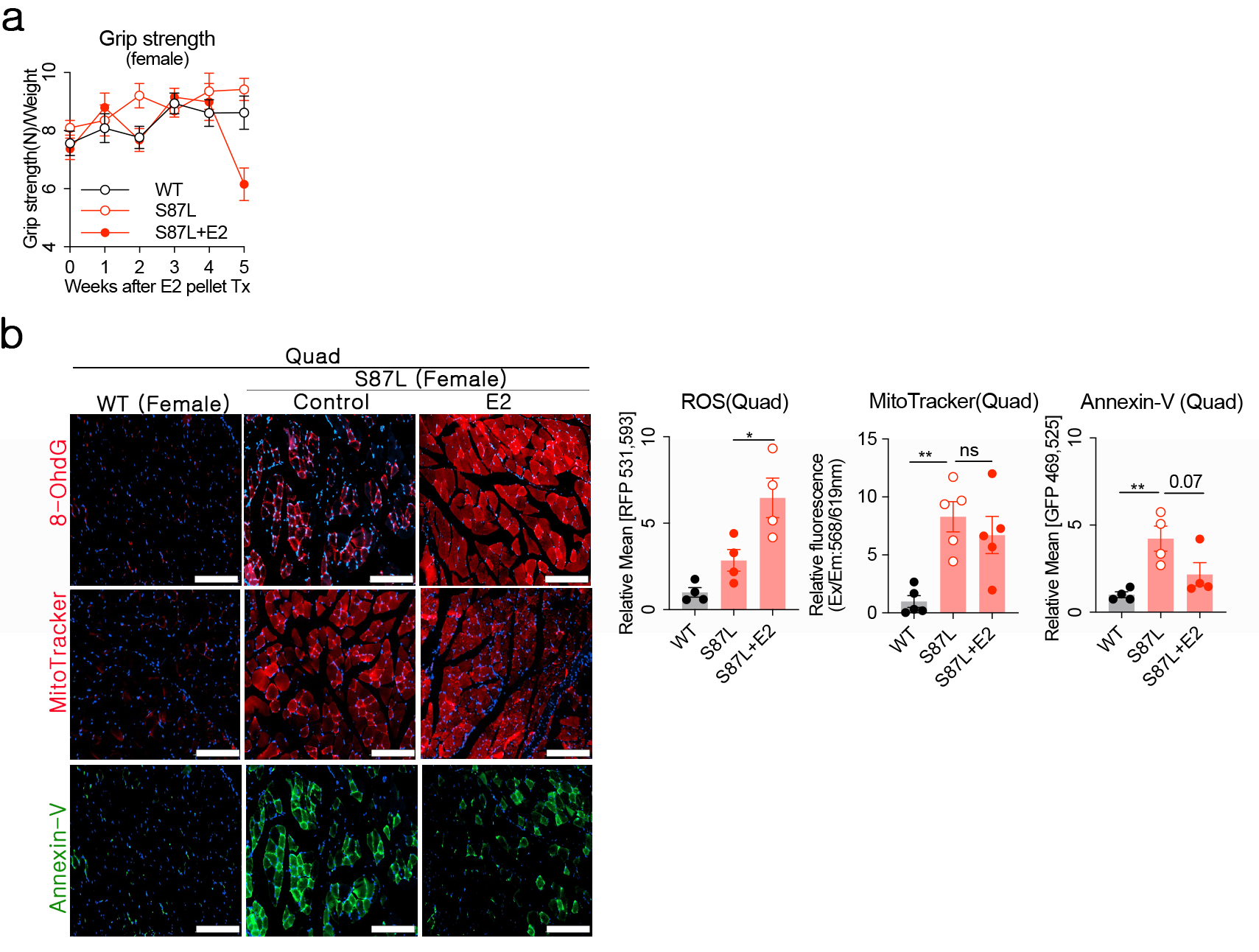


**Figure S4**. Effect of estradiol treatment on the Quad of female *Morc2a* p.S87L mice. (**a**) Grip strength was measured weekly (n = 6 per group). (**b**) Representative immunofluorescence images and quantification of 8-OHdG, MitoTracker, and Annexin-V staining in the Quad eight weeks after estradiol treatment. (white scale bar = 200 μm)

**Supplementary Table 1**. Antibody information and siRNA sequences used in this study

| Target | Sequence(5’-3’) |
| --- | --- |
| siMorc2a | GUGAAGAUGUACGAUUGAU  AUCAAUCGUACAUCUUCAC |
| siEsr1 | CAGAUAGGGAGCUGGUUC  UGAACCAGCUCCCUAUCUG |
| siEsr2 | CGACCUUUGAAUGGUGUCU  AGACACUCAAAGGUCG |
| siGPER1 | GUGAAGGUGGAAGGAGAUU  AGUCUCCUUCCACCUUCAC |

|  |  | Clone | Host | Dilution | Company | Cat.No |
| --- | --- | --- | --- | --- | --- | --- |
| 1st | Morc2a | Polyclonal | Rabbit | 1:2000(WB) | Abnova | PAB15729 |
|  | 8-OHdG | Polyclonal | Rabbit | 1:300(IF) | Biossantibodies | BS-1278R |
|  | Esr1 | 33 | Mouse | 1:1000(WB) | Invitrogen | MA1-310 |
|  | Esr2 | Polyclonal | Rabbit | 1:1000(WB) | Invitrogen | PA1-310B |
|  | GPER1  Islet1  BRN3A  VGLUT2 | Polyclonal  Polyclonal  Polyclonal  EPR21085 | Rabbit  Rabbit  Rabbit  Rabbit | 1:3000(WB)  1:100(IF)  1:100(IF)  1:200(IF) | Invitrogen  Abcam  Abcam  Abcam | PA5-28647  Ab20670  Ab245230  Ab216463 |
|  | β-actin | D6A8 | Rabbit | 1:8000(WB) | Cell signaling | 8457S |
|  | α-tubulin | 11H10 | Rabbit | 1:5000(WB) | Cell signaling | 2125S |
|  | chAT | Polyclonal | Goat | 1:200 | Chemicon | AB144P |
|  | Tuj1 | Polyclonal | Rabbit | 1:1000 | sigma | T2200 |
| 2nd | ɑ-Rabbit(biotinylated) |  | Goat |  | Vector |  |
|  | ɑ-Rabbit (HRP) |  | Goat |  | AbFRONTIER |  |
|  | ɑ-Mouse (HRP) |  | Goat |  | AbFRONTIER |  |
|  | ɑ-Goat(HRP) |  | Donkey |  | Invitrogen | A11058 |
|  | ɑ-Rabbit (HRP) |  | Donkey |  | Invitrogen | A21206 |

**Supplementary Table 2.** Clinical characteristics in individuals with *MORC2* mutation

| Patients | FC1366  (I-2, mother) | FC1366  (II-2, son) |
| --- | --- | --- |
| Gene: mutation | *MORC2* | *MORC2* |
| Nucleotide change | c.568C>T | c.568C>T |
| Amino acid change | p.R252W | p.R252W |
| Sex | Female | Male |
| Age at exam (years) | 55 | 13 |
| Ages at onset (years) | 8 | 3 |
| Disease duration (years) | 47 | 10 |
| Muscle weakness^a^ |  |  |
| Upper limb | ++ | ++ |
| Lower limb | ++ | +++ |
| Muscle atrophy | Moderate | Moderate to severe |
| Sensory impairment | Yes (V>P) | Yes (V>P) |
| Disabilities |  |  |
| CMTNSv2 | 14 | 19 |
| FDS^b^ | 3 | 4 |
| Deep tendon reflexes |  |  |
| Knee jerk | Absent | Absent |
| Ankle jerk | Absent | Absent |
| Foot deformity | Yes | Yes |
| Scoliosis | Yes | Yes |
| Ankle foot orthosis | No | Yes |
| Wheelchair bound | No | No |
| Developmental delay | No | No |
| Vocal cord paresis | No | No |
| Tremor | No | No |
| Hearing loss | No | No |
| Respiratory insufficiency | No | No |

Abbreviations: A: absent, CMT: Charcot-Marie-Tooth disease, CMTNSv2: CMT neuropathy score version 2, FDS: functional disability scale, P: pain sense, V: vibration sense.

^a^ Muscle weakness: Upper limb; +: intrinsic hand weakness 4/5 on the MRC scale, ++: intrinsic hand weakness < 4/5 +++: proximal weakness, on the MRC scale. Lower limb; +: ankle dorsiflexion 4/5 on the MRC scale, ++: ankle dorsiflexion < 4/5, +++: proximal weakness.

^b^ FDS: 0: normal, 1: normal but with cramps and fatigability, 2: inability to run, 3: walking difficulty but still possible unaided, 4: walk with cane, 5: walk with crutches, 6: walk with a walker, 7: wheelchair bound, 8: bedridden.
